# Supercrystallography-Based Decoding of Structure and Driving Force of Nanocrystal Assembly

**DOI:** 10.3390/ma12223771

**Published:** 2019-11-17

**Authors:** Xin Huang, Zhongwu Wang

**Affiliations:** Cornell High Energy Synchrotron Source, Wilson Laboratory, Cornell University, Ithaca, NY 14850, USA; xh78@cornell.edu

**Keywords:** nanocrystal assembly, supercrystal, supercrystallography, superlattice, translational and orientational ordering, driving forces, interaction and environment, materials design

## Abstract

Nanocrystal (NC) assembly appears as one promising method towards the controllable design and fabrication of advanced materials with desired property and functionality. The achievement of a “materials-by-design” requires not only a primary structural decoding of NC assembled supercrystal at a wide range of length scales, but also an improved understanding of the interactions and changeable roles of various driving forces over the course of nucleation and growth of NC superlattice. The recent invention of a synchrotron-based X-ray supercrystallographic approach makes it feasible to uncover the structural details of NC-assembled supercrystal at unprecedented levels from atomic through nano to mesoscale. Such structural documentations can be used to trace how various driving forces interact in a competitive way and thus change relatively in strength to govern the formation of individual superlattices under certain circumstances. This short review makes use of four single supercrystals typically made up of spherical, truncate, cubic and octahedral NCs, respectively, and provides a comparable description and a reasonable analysis of the use of a synchrotron-based supercrystallographic approach to reveal various degrees of translational and orientational ordering of NCs within various superlattices. In the connection of observed structural aspects with controlled environments of NC assembly, we further address how various driving forces interact each other to develop relatively changeable roles upon variation of the NC shape to respond to the nucleation and growth of various superlattices. With the guidance of such gained insights, we provide additional examples to illustrate how realistic environments are designed into delicate control of NC assembly to achieve particular interactions between NCs towards harvesting superlattice with NC translational symmetry and atomically crystallographic orientation as desired.

## 1. Introduction

Recent advances in wet synthetic chemistry have allowed the designable fabrication of monodispersive nanocrystals (NCs) with excellent control over a series of unique identities of a particle size, shape, composition and surface coating molecule [1,2,3,4,5]. With such unique identities and emergent typical properties [4,5,6], NCs are considered as easily accessible building blocks, which compose a rapidly extended library of artificial designer atoms to form a completely new type of periodic table [6,7,8,9,10,11,12]. Like the ordinary atoms which come from the traditional periodic table used in materials manipulation, NCs can be selected not only similarly but also desirably from such a man-made and addressable periodic table with the primary consideration of a distinctly encoding confinement property of individual NCs being arranged into periodically ordered structures [6,12,13]. Such NC-based periodic structures are called “superlattices” to distinguish them from the ordinary atomic lattices of crystalline solids. On the one hand, NC-assembled superlattices continue to display the unique physical and chemical properties of the designer building blocks of individual NCs as desired [6,13,14], and on the other hand, NCs within a superlattice come up with strong delocalization and electronic coupling through well-arranged and embedded interfaces, which span periodically over a largely extended space to collect additional properties [15,16,17]. These newly emergent NC-based collective properties are either dramatically enhanced or completely different from the ordinary crystals made up of atoms and molecules or both [15,16,17,18].

Extensive experiments have been performed in a variety of NC systems to discover NC-assembled superlattices and thus to expand the superlattice library [19,20,21,22,23,24,25,26]. To date, the discovering process of NC superlattice has remained primarily at a quite unpleasant and time-consuming stage, in which a random and well-known trial-and-error method is mostly used. In order to accelerate superlattice discovery and thus to achieve a designable ability of superlattice fabrication, it is extremely important and highly required to improve our understanding on superlattice formation and the underlying mechanisms/kinetics. The understanding scope not only covers the nucleation and growth pathway of NC superlattice, but is also extended to the competitive interactions and emergent unique roles of individual driving forces under environments of NC assembly.

In-situ liquid transmission electron microscopy (TEM) has been quickly emerging as a powerful tool to visualize the early scenarios of NC superlattice formation in real time [27,28]. Unfortunately, the low penetrating ability of the electron beam into a sample limits the study of NC assembly into only several NC monolayers, which are not only very thin but also confined to an unusual vacuum environment. Such a fatal limitation also exits in the TEM-based electron diffraction technique, which cannot be overcome in the study of large superlattice crystals [26]. Apparently, the insights gained cannot be applied to interpret the NC assembly that takes place under a real and very complex environment. Synchrotron X-rays have unparalleled abilities of extremely high intensity and strong sample penetration; therefore, the study of NC assembly can be extended not only to large length scales but also under various and real-world environments [29,30,31,32,33]. 

Upon the rapid development of synchrotron-based X-ray techniques, both small- and wide-angle X-ray scattering (SAXS/WAXS) can be simultaneously collected from the same volume of samples so that very reliable correlations can be made across multiple length scales to reconstruct the pathway of NC assembly over the course of the nucleation and the growth of NC superlattice and subsequent phase transformation [30,34,35,36]. However, small domains of nucleated superlattice produce only a series of powder-like X-ray scattering rings; thus, the simple data-averaging used in data processing does not provide sufficient information to decipher some subtle structural variations and the underlying roles of driving forces in the process of NC assembly [7,8,9,10,11], which are indeed extremely significant for the designable fabrication of desired superlattice. 

In order to identify the interacting roles of various driving forces, typically among which one comes to dominate the formation of a typical superlattice, a synchrotron-based supercrystallographic approach was invented and improved subsequently by a team effort at the Cornell High Energy Synchrotron Source (CHESS) of the Cornell University [37,38,39,40]. Upon the success of this unique approach for the reconstruction of the structure of NC-assembled supercrystal at an unprecedented level, we were able to combine the decoded structural details to uncover the governing play of one or several typical driving forces in the process of NC assembly, thus improving our designable ability to fabricate and engineer the next generation of functional materials used for a wide array of technologies. 

In this paper, we start with a brief description of the synchrotron-based supercrystallographic approach in Section 2, which includes the key processing protocol, unique characteristics, advantage and limitations in applications. In Section 3, we select several typical supercrystal examples as grown from various shaped NCs and show the use of synchrotron-based supercrystallography to detail the structure reconstructions and correlations at both atomic and nanometric scales with subsequent decoding of the dominate driving forces responsible for superlattice formation. In Section 4, we provide a careful analysis and make feasible correlations between structure and driving forces between various NC assemblies to derive the changes of NC superlattice and the underlying driving force as a function of the NC shape under a closely or identically controlled environment. In Section 5, we use PbS NCs as an example and describe the use of derived insights to guide an experimental design to harvest superlattices as desired. Finally, we close the paper in Section 6 with a summary and a perspective of gained insights with potential use in controlled NC superlattice fabrication and the designable processing and engineering of such large supercrystals. 

## 2. Synchrotron-Based Supercrystallography

The term of “supercrystallography” is based on the traditional crystallography and is thus aimed at resolving the structural details of NC-assembled superlattice crystal, called “supercrystal”. The application can be expanded to a scope to study supercrystals made up of small clusters or large colloidal particles, or beyond. 

In brief, SAXS and WAXS images are simultaneously collected from a single supercrystal specimen. Upon rotation of the single supercrystal specimen, the full sets of SAXS and WAXS images are collected and correlated to reconstruct the full spectra of supercrystal structures, which cover not only the translational ordering of NCs as positioned in the precisely crystallographic sites, but also the orientational alignment of atomically crystallographic planes across the arrayed NCs over a largely extended space [26,37,38,39,40]. Taking into account the size-induced dramatic weakening of X-ray scattering typically in the wide-angle range, the traditional house X-ray sources do not allow one to collect useful datasets for reasonable analysis of WAXS with correlation to SAXS. Even in the small-angle range, the collection of SAXS patterns is extremely time-consuming. Evidently, the use of synchrotron X-rays is not only critical but also highly efficient.

Rather only a computer-based *hkl*-indexing of X-ray scattering spots, this synchrotron-based supercrystallographic approach is indeed composed of several significant components/steps, including (1) the growth of large enough single supercrystals, normally >50 microns, (2) in-house construction of a portable two-circle rotation diffractometer, (3) synchronization of incident X-rays and detectors with supercrystal specimen and a rotation stage, and (4) indexing and analysis of the collected datasets of SAXS and WAXS images for structural reconstruction.

Two solution-based approaches of NC assembly are mostly used to grow large supercrystals. One employs the slow evaporation of NC-suspending solutions, and the grain sizes of growing supercrystals cover a wide range, from a few microns to several millimeters or even a centimeter. The other combines the two types of solvents, called anti-solvents, to destabilize the NC stability and thus trigger the nucleation and growth of supercrystal and accordingly, the resultant supercrystals are highly faceted and cover a broad range of grains from tens to hundreds of microns.

**Figure 1:**
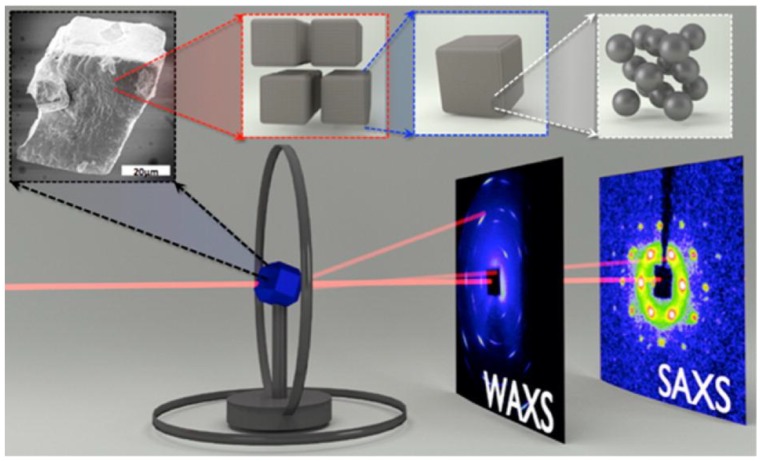
Schematics of experimental setup for the collection of both SAXS and WAXS images from the same orientations of supercrystal upon rotation of a supercrystal grain mounted on a portable two circle diffractometer. The top panel shows the full-length scales of the assembled supercrystal from atomic through nano and meso to bulk. Copyright of the American Chemical Society [38].

Upon ultimate harvest of large enough supercrystals, we begin with a primary identification of the crystallinity of supercrystal grains, which normally have sizes in the range of 50–100 microns for good scattering intensities of both SAXS and WAXS as using the CHESS facility before an upgrade. After the CHESS upgrade and at other third generations of synchrotron sources, while the X-rays are collimated down to a micron size or even smaller, the use of such brilliant X-rays allows one to reduce both grain size and exposure time for the collection of useful datasets [29,32]. Then, we select and mount an identified single supercrystal grain on a goniometer located on the two circle rotation X-ray diffractometer (Figure 1) [38]. Upon rotation of a single supercrystal specimen, the full sets of SAXS and WAXS images are collected from the same volume of supercrystal as controlled over the range of 0~180° at an angular rotation step of 1°. Using the collected datasets, the SAXS patterns are preceded by an automate indexing process to determine the translational symmetry of NC-assembled superlattice, whereas the WAXS patterns collected at all the corresponding orientations of the superlattice are implemented to determine the crystallographic orientations of NCs embedded in the defined superlattice symmetry. 

Upon structural reconstruction of NC supercrystals made up of variously shaped NCs and subsequent derivation of the encoding information, we are able to identify the dominant driving forces responsible for the formation of NC superlattices. Note that the technical details can be found in previous publications and associated supporting materials [26,37,38,39,40].

## 3. Shape-Dependent Superlattices of NC Assembles

Design and control of NC synthesis are experimentally achieved, capable of fabricating monodispersive NCs with desired shapes for various purposes, such as the study of NC assembly [41]. Extensive investigations revealed the dependence of the NC shape on the particle size [1,2,3,4,5]. While NCs are smaller than 5 nm in diameter, the shapes are mostly spherical [1,2,3,4,5]. Upon growth of NC particle, the resultant larger NCs come up with various degrees of surface truncations and are eventually developed into either cubes or octahedra [25,41,42,43,44]. In the case of one solid that has an atomic cubic structure, once NCs grow larger than 10 nm, the shape is developed to be either octahedral or cubic [25,41,42,43,44]. The factor which determines the shape transformation of NCs from a sphere to either a cube or an octahedron depends largely on how synthetic experiments are truly controlled [43,44]. 

Previous studies on spontaneous self-assemblies of spherical and anisotropic NCs have been extensively performed by various research groups and a series of superlattice symmetries have been observed accordingly [6,12,13,14,18,19,20,21,22,23,24,25,26]. While the close-packing superlattices of face-centred cubic (fcc) and hexagonal closing packing (hcp) appear to be very common in the case of spherical NCs [6,13,14,18,22,34,36,41,42,45], superlattices with recued symmetries nucleate and grow from solutions as NCs change in shape [12,26,31,38,39]. However, there are always exceptions occurring, such as the low packing density bcc in spherical NCs [34] and the high symmetrical fcc with low-orientational ordering in anisotropic NCs [21,25]. Without systematic and consistent control of experiments between various groups, there are too many unknown factors [46], which cause the formation of unusual superlattices, making the discovery of common rules to guide superlattice nucleation and growth difficult. 

To make a direct connection between nucleated NC superlattice and the shape-triggered effect, various shapes of NCs (e.g., spherical, truncate, octahedral and cubic) are synthesized and used to grow large free-standing supercrystals [37,38,39,40]. In order to minimize the impact of too many factors for a reliable comparison between the collected results [46], the assembly experiments of various shaped NCs are all controlled under an identical/very close solvent environment. In the following sections, single supercrystals made up of spherical, octahedral, cubic and truncate NCs are selected for collection of the full sets of SAXS and WAXS images, which are used to demonstrate the capability of the supercrystallographic approach for decoding structural details and analyzing the dominant driving forces in response to NC assembly. 

### 3.1. Spherical NCs

Spherical NCs are akin to electron isotropically surrounded metallic atoms. In the ordinary periodic table, metallic atoms mostly crystallize into either a face-centered cubic (fcc) or a hexagonal close packing (hcp) structure and they both have an atomic packing efficiency of 74%. It is thus expected that spherical NCs similarly self-assemble into an fcc or an hcp superlattice. Extensive studies of spherical NC assembly confirmed the above prediction [6,13,14,18,22,34,36,41,42], but there is little information on how NCs are developed orientationally of atomic crystallographic planes across each single superlattice domain of fcc or hcp. 

**Figure 2:**
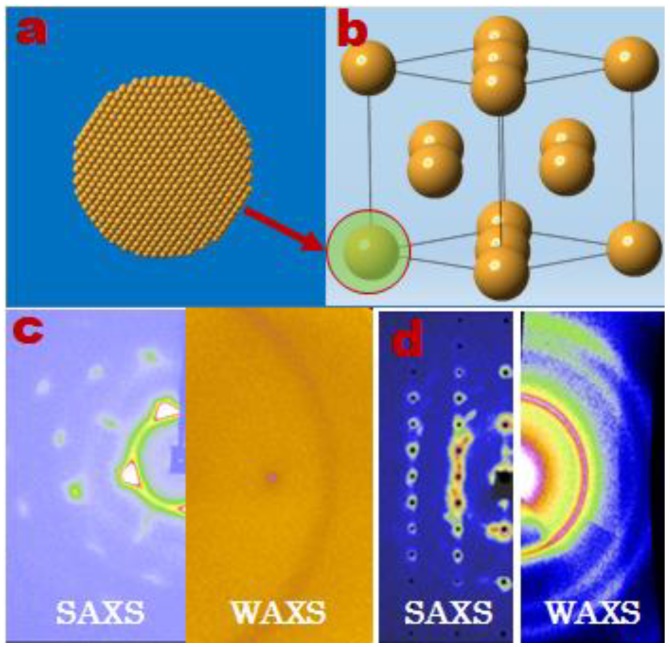
Self-assembly of (**a**) spherical Au NCs into either (**b,c**) a face-centered cubic (fcc) or (**d**) a hexagonal close packing (hcp) superlattice. Insert (**c**,**d**), left and right show SAXS and WAXS images, respectively, collected from the same volume of supercrystal at an identical orientation. Copyright of the Springe Nature group [45].

Spherical Au NCs with an average diameter of 4.4 nm are synthesized and used to grow large free-standing single supercrystals [45]. SAXS images of Au NC-assembled supercrystals (Figure 2) identify the existence of the two close-packing superlattices of fcc (Figure 2c) and hcp (Figure 2d) [45]. Examination of SAXS images collected at several typical orientations confirms the single crystallinity of free-standing supercrystal grains. Surprisingly, the free-standing single supercrystals give rise to the prototype powder-like WAXS rings. Such a texturing difference of the scattering image between SAXS and WAXS indicates that spherical Au NCs are translationally ordered but orientationally disordered. 

Each spherical NC is composed of a hard inorganic core and a soft molecular shell. Under a highly relaxing solvent environment, spherical NCs can be still reasonably considered as a series of soft nanoscale spheres. The hard sphere packing theory favors the primary formation of a close-packing superlattice, in which the configurational (positional) entropy is maximized to reduce the total free energy of NC assembly. This entropy-based energy minimization explains well not only the formation of both fcc and hcp superlattices, but also the dominant role of configurational entropic force in the self-assembly of spherical NCs. In addition, the random orientations of NCs observed in the two superlattices indicate an additional type of NC disordering which contributes extra entropy (e.g., vibrational entropy) to reduce the total free energy. The two types of entropic contributions thermodynamically promote fcc and hcp as the two energy-favorable stable superlattices. It is thus suggested that both configurational and vibrational entropies of NCs come up with close interactions to govern the self-assembly of spherical NCs into the two closely packed superlattices of fcc and hcp.

### 3.2. Octahedral NCs

An octahedral NC is terminated at surfaces by eight equivalent facets. For an ordinary solid with an atomic cubic structure, the terminating facets at NC surfaces are the low-index crystallographic planes of (111), which have a relatively a lower surface energy [47,48]. Taking into account the typical catalytic effect, the octahedral Pt_3_Ni NCs with an average edge length of 9.26 nm are used as a candidate to grow large enough single supercrystals for supercrystallographic study and subsequent analysis of the structure and driving force as well as newly emergent applications. 

**Figure 3:**
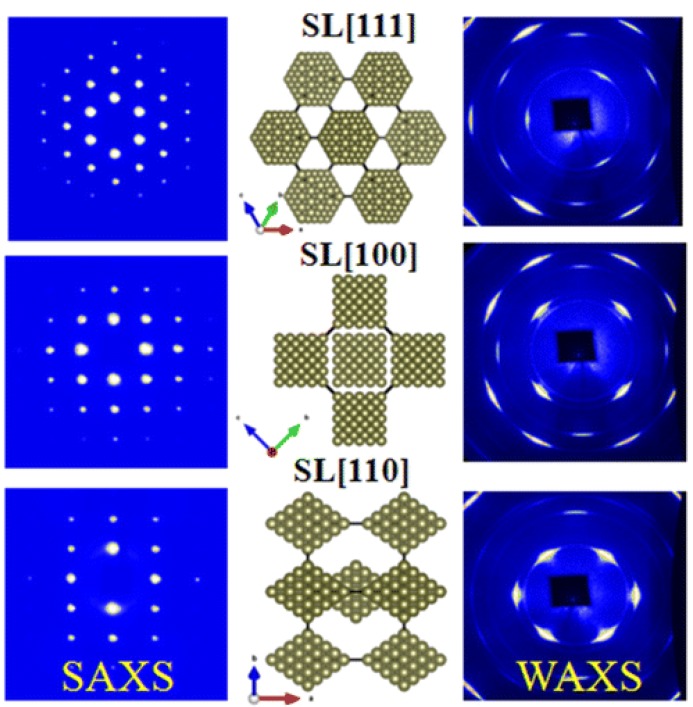
Representative SAXS and WAXS images and reconstructed structural details of single body-centred cubic (bcc) supercrystal made up of octahedral Pt_3_Ni NCs [40]. The three panels represent SAXS images of supercrystal collected from SL [111], SL [100] and SL [110] orientations (**Left**), respectively, WAXS images collected from the corresponding superlattice orientations (**Right**), and reconstructed structures at the three above-defined crystallographic directions (**Middle**). Copyright of the American Chemical Society [40].

Figure 3 shows representative SAXS and WAXS images collected from three typical crystallographic orientations and as-reconstructed structural details of single supercrystal made up of octahedral Pt_3_Ni NCs [40]. Indexing of SAXS spots determines the exclusive crystallization of a perfect body-centered cubic (bcc) superlattice from octahedral NCs. Analysis of WAXS images with correlations to SAXS defines the highly consistent crystallographic orientations of octahedral NCs within bcc. Both assembled superlattice and interior atomic lattices of NCs develop the structural relations of SL [111]//Pt_3_Ni [111], SL [110]//Pt_3_Ni [110] and SL [100]//Pt_3_Ni [100], where “SL” represents superlattice. Such structural relations at both atomic and mesoscale levels reveal the two typical arrangements of octahedral NCs of (1) vertex-to-vertex, and (2) face-to-face configurations, which are oriented along the SL [100] and SL [111] (Figure 3, middle panel) directions, respectively.

**Figure 4:**
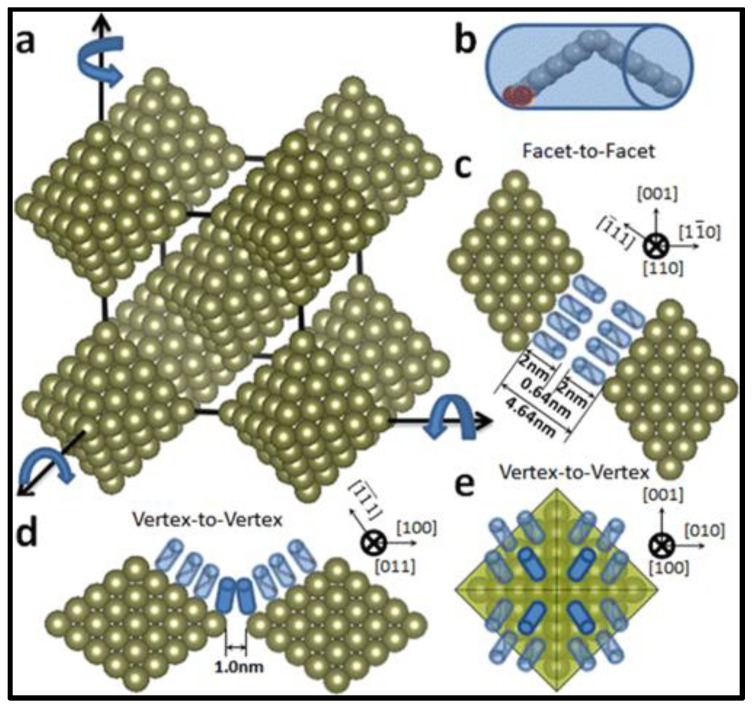
Proposed rotating model of octahedral Pt_3_Ni NCs in a bcc superlattice (**a**) and illustrations of oleic acid (OA) molecules (**b**) and arrangements of OAs in a facet-to-facet (**c**) and a vertex-to-vertex (**d,e**) configuration, respectively. OA molecules as highlighted in a solid color in (**d,e**) display a direct contact in the tip-to-tip direction of NCs. Copyright of the American Chemical Society [40].

Based on SAXS-defined cell parameters and a TEM-defined NC edge length of 9.26 nm, the inter-facet [Pt_3_Ni (111)] separation between neighboring NCs in bcc is precisely calculated as 4.64 nm (Figure 4c), whereas the inter-tip separation between neighboring NCs along the three axial directions [e.g., (001), (100) and (010)] is 1.0 nm (Figure 4d). In comparison with the double oleic acid (OA) molecular length of 4.0 nm [34], a noticeable gap of 0.64 nm (e.g., 4.64–4.0 nm = 0.64 nm) remains unfilled between the fully relaxed OA molecules stretched out from neighboring surface Pt_3_Ni (111) facets, but an apparent inter-molecular intercalation (or touching) appears between the octahedral tips of neighboring NCs. These precisely defined parameters indicate that octahedral NCs in bcc are capable of free rotation along either one of the three major superlattice axes (e.g., the tip direction of NC) (Figure 4).

The above analysis and arguments suggest that rotational entropy comes to play an additional and significant role in the self-assembly of octahedral NCs towards the formation of an open bcc superlattice. In comparison with the theoretical NC packing efficiency of 74% in fcc, the bcc reduces the packing efficiency down to 68%, which is indicative of a significant decrease of position-based configurational entropy. Thermodynamically, the formation of a new structural phase requires a reduced free energy which is lower than that of the primary structure of fcc. Therefore, the observed nucleation of the low packing bcc superlattice implies that the newly emergent rotational entropy is at least capable of compensating for the loss of configurational entropy from fcc. It is thus suggested that the competitive interactions and rational optimizations between rotational and configurational entropies are responsible for the nucleation and growth of the bcc superlattice. In other words, the shape-induced rotational entropy overplays the position-based configurational entropy, driving octahedral NCs to form an open bcc superlattice, rather than the mostly observed fcc in the self-assembly of spherical NCs [6,13,14,18,22,34,36,41,42].

### 3.3. Cubic NCs

A cubic NC (called as nanocube) with an atomic cubic structure is terminated at surfaces by six equivalent facets of (100). For consistence, we still use the term “cubic NC” to describe nanocubes in the content of this short review. As an example, cubic Pt NCs with an average edge length of 9.5 nm are used to grow single supercrystal in the collection of both SAXS and WAXS images for structural reconstruction. However, the as-synthesized cubic Pt NCs are rather perfect and always display some degree of surface truncations, giving rise to eight small (111) facets at NC surfaces.

**Figure 5:**
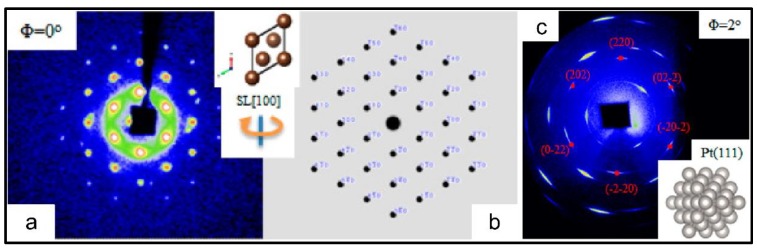
Representative SAXS and WAXS images and as-reconstructed structural details of Pt nanocube-assembled supercrystal at one typical superlattice orientation: (**a**) SAXS image along the SL (111) orientation with (**b**) simulated pattern and (**c**) corresponding WAXS image with atomic Pt (111) orientation of nanocubes. Note: an angular mismatch of 2 degrees exists between superlattice SL (111) and atomic Pt (111) orientation. Copyright of the American Chemical Society [38].

As decoded from singe supercrystal SAXS and WAXS datasets (Figure 5) [38], cubic Pt NCs crystallize in an obtuse rhombohedral (Rh) superlattice (R-3m). Using a hexagonal cell, the lattice parameters are calculated as a = b = 19.78 nm, c = 14.26 nm, and α = 120°. Using a primitive cell of rhombus, the lattice parameters are alternatively written as a = 12.37 nm and α = 106.2°. Obviously, the rhombus cell provides a straightforward view of the unique angle of obtuse Rh for easy understanding. Inside the Rh superlattice, NCs align interior atomic Pt (111) planes to Rh [111], giving rise to three types of NC alignments: (1) face-to-face, (2) edge-to-edge, and (3) corner-to-corner configurations.

A direct comparison between SAXS-defined superlattice parameters and TEM-determined cubic edge length of NCs allows one to uncover the three significant textures of NC arrangements within Rh: (1) NCs seated in the Rh (111) direction are almost touched by corners; (2) NCs in SL (111)-normal direction are interlocked together, strengthening the Rh as a highly stable mesoscale architecture; (3) a slight misorientation between NCs in the Rh (111) direction yields a noticeable angular mismatch of ~2° between Rh (111) and Pt (111) (Figure 5a,c). Additional studies of cubic NCs with variable compositions revealed the popularity of superlattice crystallization in a Rh phase. The difference between observed Rh superlattices is reflected only by a slight angular fluctuation of the rhombus cell. For example, obtuse angles of 104° and 106° were observed in two Rh superlattices made up of cubic Pt_3_Co and Fe_3_O_4_ NCs, respectively [35,39].

**Figure 6:**
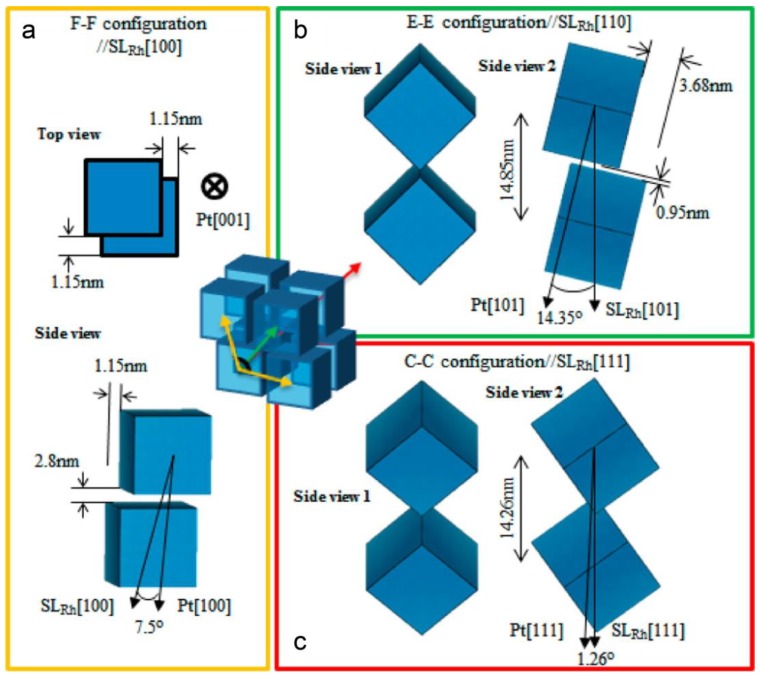
Packing configurations of cubic NCs and defined parameters of neighboring NCs in an obtuse rhombohedral (Rh) superlattice: (**a**) face-to-face, (**b**) edge-to-edge, and (**c**) corner-to-corner arrangements. Copyright of the American Chemical Society [38].

As calculated and shown in Figure 6, the facet-to-facet separation is 2.8 nm, whereas the edge-to-edge separation is 0.95 nm. Unlike the rotation-capable large freedom of octahedral NCs in bcc, the interlocking structure of NCs in obtuse Rh does not give NCs much freedom to migrate or rotate and instead, larger parallel areas and smaller facet-to-facet separation are developed between neighboring NCs, thus resulting in a dramatic increase of conformational entropy of surface molecules interfaced between cubic NCs. An in-situ SAXS study on cubic Fe_3_O_4_ NC assembly [35] revealed a close structural linkage between obtuse Rh and bcc, indicative of the reminiscence of bcc-based low packing configuration and the resulting low configurational entropy. Such typical structural features suggest that a shape-induced enhancement of conformational entropy from the interactions of surface molecules comes to compensate for the loss of configurational and rotational entropies of hard NCs, thus serving as the dominant stimulus to govern the self-assembly of cubic NCs into an obtuse Rh superlattice.

### 3.4. Truncate NCs

Truncate NCs can be considered as a series of polyhedral intermediates between spherical and octahedral ones. For a solid with an atomic cubic structure, a truncate NC is terminated at surfaces by eight (111) and six (100) facets. One of our previous studies made use of truncate PbS NCs to grow a single supercrystal, which was thus used to reconstruct translational and orientational ordering of truncate NCs in an assembled superlattice [37].

**Figure 7:**
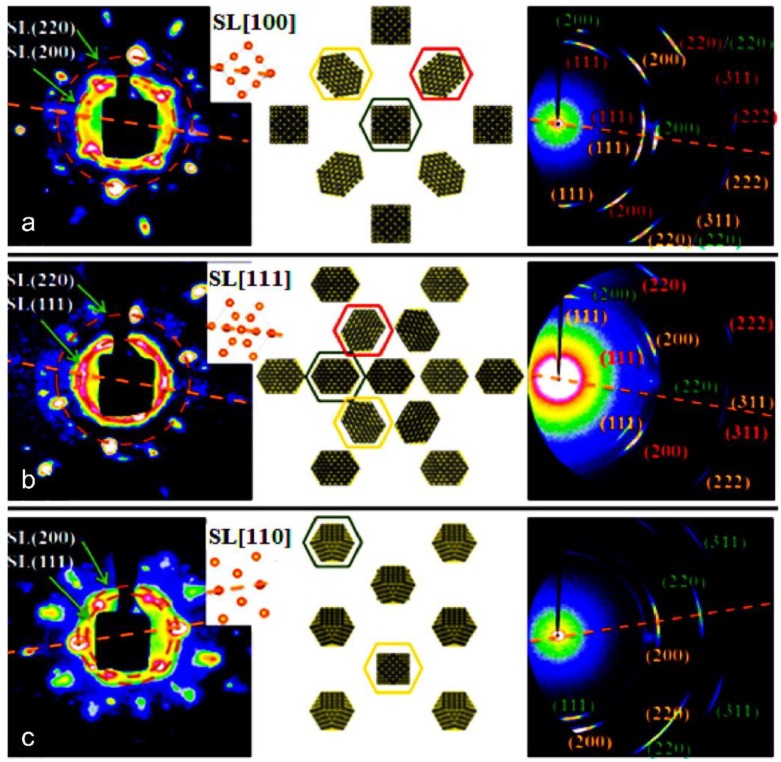
Structural reconstruction of truncate PbS NC assembled supercrystal as projected from SL [100] (**a**), SL [111] (**b**), and SL [110] (**c**) orientations, respectively. Three panels include: (**Left**) SAXS patterns with inset showing the superlattice projection; (**Middle**) structure and orientations of truncate NCs at typical crystallographic directions; and (**Right**) WAXS images with Miller indices. Note: (1) the dotted lines and circles guide comparison; (2) the various colors show differently oriented NCs in superlattice that yield X-ray scattering spots as marked in the same colors. Copyright of the American Chemical Society [37].

**Figure 8:**
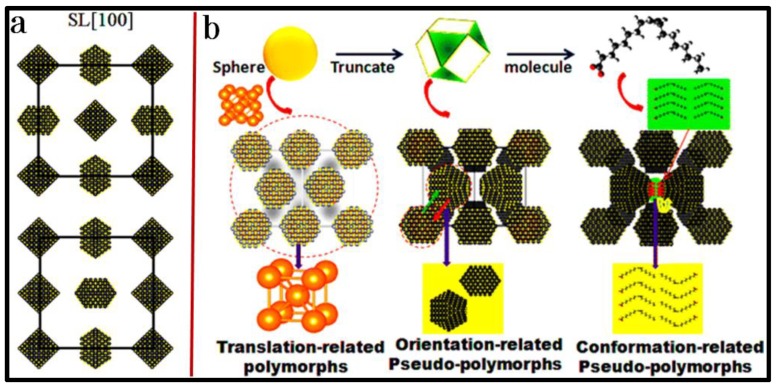
(**a**) Two non-degeneration shape-based superlattice pseudo-polymorphs of fcc superlattice made up of truncate PbS NCs as projected in SL [100], which yield identical sets of SAXS and WAXS patterns. The top and bottom of inset (**a**) include a *C*_4*h*_ and *C*_2*h*_ symmetry, respectively; (**b**) three types of fcc superlattice pseudo-polymorphs, including translation-, orientation- and conformation-related ones. Copyright of the American Chemical Society [37].

Figure 7 represents three typical SAXS and WAXS images and the as-reconstructed structure of the supercrystal made up of truncate PbS NCs [37]. Apparently, truncate PbS NCs crystallize into an fcc superlattice, giving rise to the two groups of crystallographic orientations of NCs inside. As shown in the middle panel of Figure 7, NCs at face-centers have different crystallographic orientations than those at the corners. Additional analysis reveals different groups of NC orientations, which can produce identical sets of SAXS and WAXS patterns, as observed (Figure 8a), allowing one to derive a series of NC-based superlattice polymorphs (Figure 8b). In combination with NC shape configuration and surface molecular conformation, there are three types of superlattice pseudo-polymorphs, including translation-, orientation- and conformation-based pseudo-polymorphs (Figure 8b) [37]. Similarly to but much more complicated than the structural polymorphs observed and well defined in the ordinary atomic structures, such newly emergent superlattice polymorphs dramatically enrich the library of NC-based superlattice, which provides additional toolkits for the designable fabrication of functional materials with increased structural complexity.

In connection with entropic forces which govern the NC assembly, NCs tend to pack tightly to maintain a higher configurational entropy, favoring the formation of an fcc superlattice. Once NCs are truncated, a shape-triggered crystallographic orientation appears in the NC assembly and starts to make additional contribution to orientational entropy. The two types of entropic forces interact and drive truncate NCs to form a series of very complex fcc superlattices in terms of achievement of various local energy minima. Upon modification of the NC shape or assembly environment or both, the strength of the competitive interactions between configurational and orientational entropies is either enhanced or weakened, accordingly creating various degrees of orientational ordering in fcc.

## 4. Dependence of Driving Forces and Superlattices on the NC Shape

Extensive experiments have explored the formation of various superlattices and underlying driving forces [6,12,13,14,18,19,20,21,22,23,24,25,26,34,36,41,42,45]. In the case of spherical NCs with soft molecular decoration at the surfaces, various superlattices are observed as either NC size or solution environment changes. While the relative length ratio of interior NC core to surface coating molecules explains the size-dependent variation of superlattice [49], the preservation or loss of surface coating molecules explains the environment-dependent variation of superlattice [50]. In both cases, the assembly process involves a slight development of NC shape in terms of a deformation of surface ligand shell or an exposition of truncate cores, which implies the significant play of the NC shape on NC assembly and the developed superlattice.

With experimental control of the NC assembly under a close or identical environment, the structural reconstructions of NC supercrystals made up of various shaped NCs provide fundamental details to recognize the shape-dependent variation of NC assembly in both superlattice symmetry and orientational ordering. Thus, the structural information derived from NC supercrystals enables one not only to understand the competitive interactions between various entropic forces, but also to distinguish the unique and changeable roles of individual driving forces over the course of NC assembly towards ultimate formation of various typical superlattices.

**Figure 9:**
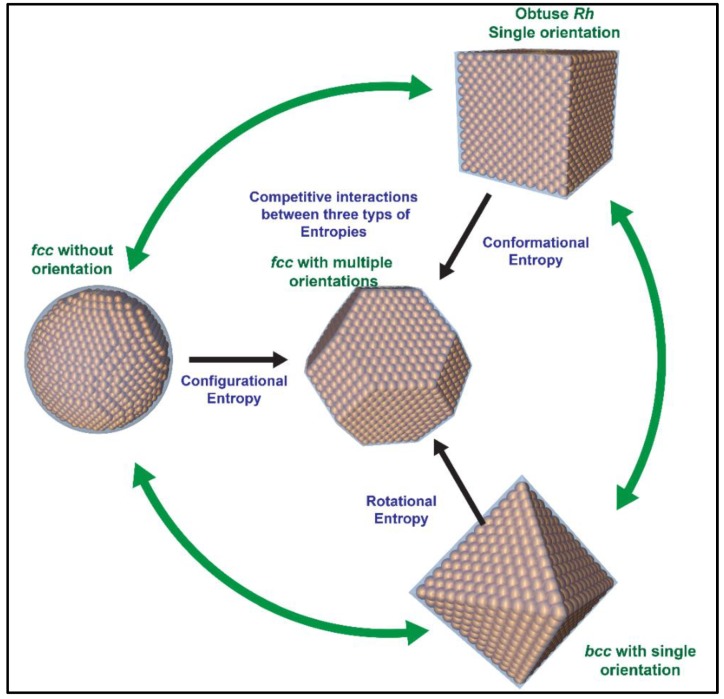
Dependence of various entropic forces on the NC shape over the course of NC assembly towards the formation of typical superlattices, which display various degrees of translational and orientational ordering of NCs.

Figure 9 summarizes up the derived variation of three major entropic forces over the process of NC assembly upon change of the NC shape, which lays foundation not only to understand the nucleation and growth of various superlattices, but also to guide the designable fabrication of NC superlattice with desired NC translation and atomic crystallographic orientation. As mentioned above, NC assembles are all controlled under very close environments, including (1) homogeneous suspensions of NCs in a good solvent of toluene or hexane and (2) slow evaporation of NC suspended solution in a parafilm-sealed glass container. As a result, the collected information of the superlattice symmetry and the driving force from various NC supercrystals can be reliably correlated to yield invaluable insights into shape-dependent changes of NC interaction, driving force, superlattice symmetry and orientational ordering.

Starting with spherical NCs, the position-based configurational entropy serves as the dominant driving force, pushing NCs to form either a single fcc, hcp or both. Once NCs change in shape, a newly emergent directional entropy begins to play a significant role in the formation of the NC superlattice. As the NC shape is close to an octahedron, the shape-triggered rotational entropy comes to dominate or even overplay the position-based configurational entropy, and accordingly, an open bcc superlattice is exclusively formed. Alternatively, NCs can be also shaped into cubes and correspondingly, NC assembly starts to precede with a similar but slightly different way as that in octahedral NCs. Cubic NCs tend to reduce the configurational packing efficiency and instead, to maximize the shape-based directional entropy. As opposed to the octahedral case, cubic NCs try to align their flat facets to increase the inter-NC interacting areas and as a result, a ligand-based conformational entropy is maximized to compensate for the loss of NC-based configurational and rotational entropies. Ultimately, the newly developed/achieved minimization of total free energy favors the formation of an obtuse Rh superlattice, which is structurally close to the bcc but orientationally enhances atomically crystallographic planes. It is thus understandable that NCs with an equivalent ratio of (111) to (100) truncation areas continue to form an fcc superlattice, as observed and predicted, in which the extent of orientational ordering is weaker than those in cube-based Rh and octahedron-based bcc, but much stronger than that in sphere-based fcc. In addition, it is also reasonable to observe a mixture of fcc and bcc or a single bcc in truncate NCs, if a feasible assembly environment is well designed and delicately controlled.

## 5. Design of Assembly Environments towards the Collection of Desired Superlattices

The goal of controlled materials fabrication is ultimately to achieve designing ability, which is based on the primary discovery of useful designer rules by experiments. To this end, computational simulation has the potential to provide fast prediction and valuable guidance [8,9,10,11], but it still requires large input from the experimental side to test the reliability. As a significant step, we gained systematic insights from the supercrystallographic studies described in Section 4. With such insights, we are able to design feasible assembly environments to achieve specific NC interactions and thus harvest the superlattice as desired. The design of an assembly environment with desired NC/ligand interactions can be effectively achieved by either a single or multiple controls of NC concentration, dispersing solvent, additive molecule and so on.

**Figure 10:**
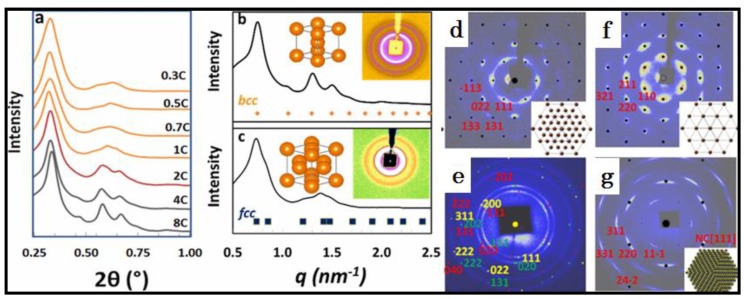
Controlled assembly of PbS NCs with intermediate ratios of surface truncations into single fcc and bcc superlattices, respectively. SAXS patterns showing (**a**) the concentration-dependent formation of 7.5 nm PbS NC superlattice into a mixture of or single (**b**) bcc and (**c**) fcc upon drop-casting of NC-suspending toluene; (**d**) SAXS and (**e**) WAXS images collected from a large single fcc supercrystal made upon slow evaporation of 6.7 nm PbS NC suspensions in a toluene solution with extra oleic acids; (**f**) SAXS and (**g**) WAXS images collected from a large single supercrystal obtained from an anti-solvent diffusion of isopropanol into 6.7 nm PbS NC-suspending toluene. Note: 1C = 17.5 mg/mL. Copyright of the American Chemical Society [46,55].

Among a large variety of NC systems, PbS NCs have been extensively used to study the spontaneous NC self-assembly, and various superlattices have been observed accordingly, including fcc, bcc and a series of intermediate ones, such as the tetragonal superlattice [18,25,41,46,51,52,53,54,55]. Instead of covering many NC systems with too many details to distract attention, only PbS NCs with intermediate ratios of surface truncations are used as an example here to detail how the design and control of typical environments are experimentally made to activate specific NC interactions towards the ultimate harvest of a single superlattice of fcc or bcc at both small and large scales as desired (Figure 10) [46,55].

### 5.1. Single Face-Centered Cubic (FCC) Superlattice

The exclusive formation of single fcc requires the achievement of the dominant enhancement of configurational entropy and weakening the strengths of both directional/conformational and rotational entropies under certain environments of NC assembly. Because of the truncation feature of PbS NCs with a 7.5 nm in diameter, a designer environment has to be able to dramatically weaken or completely eliminate the shape-based influence on NC interactions. As one easy way, truncate NCs are suspended in a good solvent, such as toluene, with a very low concentration (normally smaller than 17.5 mg/mL) so that the surface molecules are fully relaxed in solvent, activating the core-shell NCs as spherical units to form a position-maximized superlattice of fcc (Figure 10a,c) [46]. Under such a dilute NC environment, one apparent drawback is the formation of only small fcc superlattice grains, as confirmed by the observed powder-rings in inset Figure 10c [46].

Large growth of fcc supercrystal can be feasibly achieved by increasing NC concentration and introducing an extra amount of OA molecules in solvent. With additional OA molecules, the spherical feature of NCs can be dramatically enhanced so that the shape effect is minimized to play only a very small role in the process of NC assembly. Once the molecular control of assembly environment assures an exclusive nucleation of the fcc superlattice, a supplementary increase of NC concentration in solvent provides additional amounts of NCs, allowing small nucleated fcc seeds to grow larger. However, an OA-triggered screening effect appears to weaken the strength of the direct interactions between inorganic NC cores so that the extent of orientational ordering is largely reduced in the nucleated fcc superlattice (Figure 10d,e) [55]. With a similar experimental control, large fcc supercrystals with multiple groups of orientational ordering were observed by various research groups [51,56], confirming the reliability of the derived guiding rules.

### 5.2. Single Body-Centred Cubic (BCC) Superlattice

Designable assembly of truncate PbS NCs into single bcc can be made by increasing NC concentration or decreasing the amount of surface-coating molecules in solvent. As one typical example which uses truncate PbS NCs with an average diameter of 7.5 nm [46] and am NC concentration larger than 70 mg/mL in toluene, a single bcc forms exclusively (Figure 10a,b). The continuous increase of NC concentration not only dramatically improves the crystallinity of bcc supercrystal, but also largely increases the size of supercrystal grain. Alternatively, the same effect can be activated by reducing the amount of surface coating molecules (e.g., strong washing) so that NCs at a reduced concentration can still crystallize in a bcc superlattice, but the supercrystal grains are very small.

### 5.3. Alternative Environmental Designs

In addition to the control of NC concentration and surface molecules, environmental designs can be also made by tuning other experimental components to achieve desired NC interactions towards the ultimate harvest of single fcc or bcc superlattice. In comparison with toluene, the use of hexane or chloroform as a dissolving solvent can reduce the NC solubility [46]. Indeed, this simple solvent-switching approach serves as an alternative way to trigger an indirect increase of NC concentration, which thus induces the assembly of NCs into a single bcc superlattice.

In addition, the anti-solvent approach, such as the diffusion of ethanol into toluene through a soft liquid–liquid interface [35], can be used to trigger the gradual increase of the NC concentration over a period of a slow diffusion process, accordingly causing the nucleation and growth of a bcc superlattice. Using the same batch of truncate PbS NCs with 6.7 nm, large supercrystals are grown by a slow diffusion of isopropanol into toluene [55]. While SAXS reveals the single crystallinity of bcc superlattice (Figure 10f), WAXS identifies highly orientational ordering of NCs (Figure 10g), consistently confirming the prediction derived from a large single bcc superlattice crystal (Figure 3) [40].

### 5.4. Computational Design

Computational advances enable the simulation of NC assembly with large numbers of colloidal NCs [8,10,57]. In particular, recent computational studies have incorporated chemical constrains of surface and solvent molecules and surface truncation ratio into the simulation of NC assembly [7,58,59]. Upon the computational discovery of new superlattices, such chemical constrains become much more easily recognizable by experimentalists to make precise designs of environments and interactions to collect desired superlattices [7,48].

In the case of truncate PbS NCs, recent computations have focused on the interactions between surface coating molecules and solvents with truncate NCs and provided two detailed kinetic superlattice diagrams of cuboctahedral and truncated-octahedral PbS NCs as functions of surface ligand length and solvent parameter [7]. Based on the phase diagrams, one could delicately play the defined parameters using various types of carbon-hydrogen ligands and solvents in experiments to harvest single superlattice of either bcc or fcc or others. Witnessing the agreement between computational predictions and experimental discoveries [34,36,46,51,52,53,54,55,56] and the newly emergent approach of machine learning [60], computational design will certainly come to serve as an increasingly important tool in the future design and fabrication of desired NC superlattices with tailored properties for applications.

## 6. Summary and Outlook

NC assembly involves complex interactions of a variety of driving forces with relatively changing strengths as a consequence of the variation of size and shape of NCs and assembly environment. Once NC assembly environments are identically controlled (e.g., the same conditions of solvent, evaporation and so on), the strengths of various driving forces also change relatively, along with the variation of the NC size and shape to impact the nucleation and growth of NC supercrystal, thus determining the translational symmetry and crystallographic orientation of NC assembly. The synchrotron-based X-ray supercrystallographic approach has the unique advantages of not only resolving the structural details of NC supercrystal at an unprecedented level, but also determining the competitive interactions between various driving forces and the emergent role of individual ones over the process of NC assembly. With such decoded structural details and gained insights, one typical and effective environment of NC assembly can be designed to activate specific NC interactions, which come to play and guide NC assembly into desired superlattice.

Spherical NCs prefer to form a close packing superlattice of fcc or hcp or both, in which the driving force is dominated by a position-based configurational entropy. Upon the development of the NC shape into a cube or an octahedron, the shape-based directional or rotational entropy competes with the position-based configurational entropy, thus driving NCs to form an obtuse Rh or a bcc superlattice. These two low-symmetry superlattices display higher levels of orientational ordering. Once NCs become truncate and eventually have an intermediate shape in-between the two end shapes of a sphere and a cube (or octahedron), they start to self-assemble into either one of fcc, bcc and obtuse Rh, in which the degrees of orientational ordering differ from one to another.

Translational symmetry of NC assembly and atomically crystallographic orientation between NCs can be desirably tuned by a primary design of assembly environment and NC interaction. Without any additional change of environmental elements, the decrease of NC concentration and increase of surface coating molecules in solvent can be delicately controlled of magnitude to enhance the strength of configurational entropic force, thus achieving an exclusive formation of fcc superlattice with reduced degrees of orientational ordering. Inversely, the increase of NC concentration and de-coating of surface molecules from NCs can be controlled of magnitude to enhance the strength of the directional entropic force, thus achieving a final collection of superlattices with a reduced packing density and enhanced orientational ordering.

The achievement of environmental design with specific NC interactions allows one to grow a large single supercrystal with desired superlattice symmetry and the expected type/degree of orientational ordering of crystallographic planes. Once the large single supercrystals are harvested, one can take advantage of the unique structural features of translation symmetry and orientational ordering not only to uncover newly emergent collective properties, but also to build useful superlattice/orientation–property–functionality relations. In the case of materials development, a designable processing approach can be executed by application of an external stimulus on the pre-defined superlattice orientation of supercrystal to develop advanced materials with a new type of mesoscale architectures. As witnessed in recent pressure-processing studies on NC supercrystals [34,61,62,63,64,65,66], it can be foreseen that this superlattice-based designable processing of materials will be certainly opening up a window for the designable fabrication of the next generation of advanced materials with an increased structural complexity and improved functionality [67,68].
